# Transcriptome and chemical analyses revealed the mechanism of flower color formation in *Rosa rugosa*

**DOI:** 10.3389/fpls.2022.1021521

**Published:** 2022-09-23

**Authors:** Yiting Wang, Shaopeng Li, Ziqi Zhu, Zongda Xu, Shuai Qi, Shutang Xing, Yunyan Yu, Qikui Wu

**Affiliations:** ^1^ Shandong Provincial Research Center of Demonstration Engineering Technology for Urban and Rural Landscape, College of Forestry, Shandong agricultural University, Tai’an, China; ^2^ School of Landscape Architecture, Beijing Forestry University, Beijing, China; ^3^ College of Horticulture Science and Engineering, Shandong Agricultural University, Tai’an, China

**Keywords:** *Rosa rugosa*, flower color, transcriptome, anthocyanin, cyanidin, peonidin

## Abstract

*Rosa rugosa* is a famous Chinese traditional flower with high ornamental value and well environmental adapt ability. The cultivation of new colorful germplasms to improve monotonous flower color could promote its landscape application. However, the mechanism of flower color formation in *R. rugosa* remains unclear. In this study, combined analyses of the chemical and transcriptome were performed in the *R. rugosa* germplasms with representative flower colors. Among the identified anthocyanins, cyanidin 3,5-*O*-diglucoside (Cy3G5G) and peonidin 3,5-*O*-diglucoside (Pn3G5G) were the two dominant anthocyanins in the petals of *R. rugosa*. The sum content of Cy3G5G and Pn3G5G was responsible for the petal color intensity, such as pink or purple, light- or dark- red. The ratio of Cy3G5G to Pn3G5G was contributed to the petal color hue, that is, red or pink/purple. Maintaining both high relative and high absolute content of Cy3G5G may be the precondition for forming red-colored petals in *R. rugosa*. Cyanidin biosynthesis shunt was the dominant pathway for anthocyanin accumulation in *R. rugosa*, which may be the key reason for the presence of monotonous petal color in *R. rugosa*, mainly pink/purple. In the upstream pathway of cyanidin biosynthesis, 35 differentially expressed structural genes encoding 12 enzymes co-expressed to regulate the sum contents of Cy3G5G and Pn3G5G, and then determined the color intensity of petals. *RrAOMT*, involved in the downstream pathway of cyanidin biosynthesis, regulated the ratio of Cy3G5G to Pn3G5G *via* methylation and then determined the color hue of petals. It was worth mentioning that significantly higher delphinidin-3,5-*O*-diglucoside content and *RrF3’5’H* expression were detected from deep purple-red-flowered 8-16 germplasm with somewhat unique and visible blue hue. Three candidate key transcription factors identified by correlation analysis, *RrMYB108*, *RrC1*, and *RrMYB114*, might play critical roles in the control of petal color by regulating the expression of both *RrAOMT* and other multiple structural genes. These results provided novel insights into anthocyanin accumulation and flower coloration mechanism in *R. rugosa*, and the candidate key genes involved in anthocyanin biosynthesis could be valuable resources for the breeding of ornamental plants in future.

## Introduction

Flower color is one of the main quality traits of ornamental plants, which is determined by the content and type of anthocyanins. Anthocyanins, a class of secondary metabolites with the different substituents on the B ring of flavonoid basic skeleton, are important water-soluble pigments accumulated in vascular plants widely (Tanaka et al., 2008; Landi et al., 2015). So far, more than 700 kinds of anthocyanidins have been found in plants, which mainly derive from six anthocyanidin aglycones, i.e., pelargonidin, cyanidin, peonidin, delphinidin, petunidin, and malvidin (Jaakola, 2013). Generally, pelargonidin and cyanidin provide red pigment to flowers and fruits, peonidin makes great contributions to purple-red color of plant tissues, while delphinidin, petunidin and malvidin are responsible for blue and bluish violet color (Khoo et al., 2017). However, the relationship between anthocyanin accumulation and petal coloration varies among different species. The anthocyanin coloration can be consistent among different plant species. For example, cyanidins are the main anthocyanins responsible for the pink and red petals of *Camellia japonica* and *Prunus persica* (Cheng et al., 2014; Fu et al., 2021). In addition, the anthocyanin coloration can also show species specificity. For instance, delphinidins are the dominant anthocyanins in most plants with pure blue petals, whereas the petals of transgenic *R. hybrida* with a high percentage of delphinidins (up to 95%) are not as pure blue as any other plants (Katsumoto et al., 2007).

Anthocyanins biosynthesis process is relatively conserved and have been studied in many seed plants (Shoeva et al., 2017; Nabavi et al., 2020). The biosynthetic pathways, using phenylalanine as substrate, requires a series of catalytic enzymes. Firstly, phenylalanine is catalyzed to form cinnamic acid under the action of phenylalanine ammonia-lyase (PAL, EC:4.3.1.24). Secondly, dihydroflavonols were generated by a series of enzymes, such as cinnamate-4-hydroxylase (C4H, EC:1.14.14.91), 4-coumarate-CoA ligase (4CL, EC:6.2.1.12), chalcone synthase (CHS, EC:2.3.1.74), chalcone isomerase (CHI, EC:5.5.1.6), flavanone 3-hydroxylase (F3H, EC:1.14.11.9), flavonoid-3’-hydroxylase (F3’H, EC:1.14.13.21) and flavonoid-3’5’-hydroxylase (F3’5’H, EC:1.14.14.81), which is a key step in the metabolism of flavonoids. The performance of F3’H and F3’5’H leads to form dihydroquercetin and dihydromyricetin with different hydroxylation pattern of dihydrokaempferol and then promote the biosynthesis of cyanidin and delphinidin (Zhuang et al., 2019). Subsequently, colored anthocyanins are formed by dihydroflavonol 4-reductase (DFR, EC:1.1.1.219), anthocyanin synthase (ANS, EC:1.14.20.4), and modified by glycosylation, methylation, and acetyltransferase under the actions of glycosyl transferases (GT, EC:2.4.1.-), methyl transferase (MT, EC:2.1.1.-), and acyl transferase (AT, EC:2.3.1.-), respectively (Koes et al., 2005; Jaakola, 2013; Iaria et al., 2016).

By binding to the promoter regions of structural genes, some transcription factors (TFs) are studied the effect on the synthesis of anthocyanins, such as MYB, bHLH, WD, bZIP, and MADS-box (An et al., 2017; Lu et al., 2018; Jian et al., 2019; Khan et al., 2022). Among them, the regulatory function of MYB, bHLH, and WD40 families are well stablished, which can play regulatory roles alone or by consisting the MBW (MYB-bHLH-WD) protein complex (Ramsay and Glover, 2005; Feng et al., 2020). MYB TFs are generally considered to be the most critical TFs for the synthesis of plant anthocyanins, with a large number of R2R3-MYB TFs being isolated. Most of these TFs play positive regulatory roles in anthocyanin biosynthesis, but few of them play negative regulatory roles (Espley et al., 2009; Butelli et al., 2012; Ni et al., 2020).

*Rosa rugosa* is a famous Chinese traditional flower with aromatic, cold resistance, drought resistance, pest resistance, salt and alkali resistance (Chen et al., 2021). But so far, *R. rugosa* has not been widely used as an ornamental plant because of its monotonous color. Except four white-flowered *R. rugosa* cultivars, more than 40 other cultivars and all wild germplasms are pink- and purple-flowered, and there is a lack of excellent cultivars with novel flower colors such as red, orange, and blue. Therefore, it is important to elucidate the mechanism of flower color formation in *R. rugosa* and breed new cultivars with novel flower colors on this basis. At present, there are few reports on the mechanism of flower color formation in *R. rugosa*, only involving three cultivars with pink and white flowers. Zhang et al. (2015) studied that peonidins were the main composition that determined the petal color of *R. rugosa* ‘Zi zhi’ (deep pink-flowered cultivar). Sheng et al. (2018) found that there was almost no anthocyanin in the petals of *R. rugosa* ‘Bai Zizhi’ (white-flowered cultivar), and there were large amount of peonidins in the petals of *R. rugosa* ‘Fen Zizhi’ (light pink-flowered cultivar) and ‘Zi zhi’. Moreover, 172 *R. rugosa* germplasms were clustered into seven categories by the petal chroma values and the anthocyanin composition analysis of 21 germplasms from different groups showed that the anthocyanin types were similar but the contents were different (in process). Overall, it’s hard to explore the mechanism of flower color formation in *R. rugosa* comprehensively by the previous studies, especially for the new colored germplasms.

In this study, we explored changes in anthocyanin type, anthocyanin content and related gene expression in the petals of different *R. rugosa* germplasms with representative flower colors. The aims of the study were to: (1) confirm the type and content of anthocyanin in the petals of *R. rugosa*; (2) identify functional structural genes and TFs involved in anthocyanin biosynthesis; (3) explore the metabolic pathways and the mechanism of color formation in *R. rugosa* petals. This study can lay an important foundation for further comprehensive and in-depth interpretation of the flower coloration mechanism in *R. rugosa*.

## Materials and methods

### Plant materials

The plants of *R. rugosa* and *R. hybrida* used in this study were planted in Rose Germplasm Nursery, Forestry Experimental Station of Shandong Agricultural University, Tai’an, China (36°10′15″ N, 117°09′25″ E), where they grew under nature conditions. In 2021, three *R. rugosa* germplasms with representative flower colors (red: 7-23; pink: 8-37; purple-red: 8-16) were selected for ultra-performance liquid chromatography tandem mass spectrometry system (UPLC-MS/MS) and transcriptome analysis. At 7:00 a.m. to 7:30 a.m. on May 2^nd^, the petals at half-opening stage from three individual plants of each germplasm were sampled and pooled, immediately frozen in liquid nitrogen and then stored at –80°C. Three replicates were set for each germplasm. In 2022, six *R. rugosa* germplasms (red: 7-23, 9-12; pink: 8-37,6-30; purple-red: 8-16, 4-50) and six *R. hybrida* cultivars (red: ‘smile’, ‘La Sevillana Plus’, ‘Alcantara’; pink: ‘Carefree Wonder’, ‘Rhapsody in Blue’, ‘Pretty Sunrise’) with representative flower colors were selected for high performance liquid chromatography (HPLC) and gene expression patterns analysis. At 7:00 a.m. to 7:30 a.m. on May 4^th^, the petals were sampled, pooled, frozen and stored by the same way as in 2021.

### UPLC-MS/MS analysis

The anthocyanin types and contents in the petals of three *R. rugosa* germplasms were performed *via* an UPLC-MS/MS by Metware Biotechnology Co., Ltd. (Wuhan, China). A total of 50 mg grounded petal samples were extracted with 0.5 mL methanol/water/hydrochloric acid (500:500:1, *v/v/v*). The extract was vortexed for 5 min and ultrasound for 5 min, and then centrifuged for 3 min (12,000 g, 4°C). The residue was re-extracted under the same conditions. The supernatants were filtrated through a membrane filter (0.2 μm) and then stored injection bottles. Anthocyanins contents were detected by MetWare (http://www.metware.cn/) based on the AB Sciex QTRAP 6500 LC-MS/MS platform. The LC separation was performed using a Waters ACQUITY BEH C18 (1.7 µm, 2.1 mm* 100 mm) at a flow rate of 0.35 mL min^-1^ and a column temperature of 45°C with 2 μL injection volume. During LC process, mobile phase A was water with 0.1% formic acid (*v*/*v*) and mobile phase B was methanol with 0.1% formic acid (*v*/*v*). The separation was performed using the following gradient program: going from 5% to 50% B in 6 min, from 50% to 95% in 12 min, at 95% for 2 min, then declining from 95% to 5% in 0.5 min and at 5% for 2 min.

The MS data were acquired on a triple quadrupole-linear ion trap mass spectrometer (QTRAP) equipped with an electrospray ionization (ESI) Turbo Ion-Spray interface, operating in positive ion mode and controlled by Analyst 1.6.3 software (Sciex). The ESI source operation parameters were as follows: ion source, ESI+; source temperature, 550°C; ion spray voltage, 5500 V; curtain gas, 35 psi. Anthocyanins were analyzed using scheduled multiple reaction monitoring (MRM). MultiQuant 3.0.3 software (Sciex) was used to quantitatively analyze the pigment contents according to the retention time (RT) and peak pattern of standard substances (**Supplementary Table 1**). Mass spectrometer parameters including the delustering potentials (DP) and collision energies (CE) for individual MRM transitions were done with further DP and CE optimization. A specific set of MRM transitions were monitored for each period according to the metabolites eluted within this period.

### HPLC analysis

The contents of Cy3G5G and Pn3G5G in the petals of six *R. rugosa* germplasms and six *R. hybrida* cultivars were measured by HPLC on a Shimadzu SPD-10A platform. A total of 200 mg grounded petal samples were extracted with 6 mL ethyl alcohol/water/hydrochloric acid (500:300:1, *v/v/v*) under dark conditions for 24 h. The filtered extract was stored for further analysis. Refer to previous study (Sheng et al., 2018), the analysis of anthocyanin combined with visible ultraviolet absorption features at 520 nm were conducted for anthocyanins with Cy3G5G and Pn3G5G as standard substances (Solarbio, China).

### Transcriptome analysis

The petal samples of the same three *R. rugosa* germplasms were used for transcriptome analysis according to previous study (Wu et al., 2020). Total RNA was extracted using Total RNA isolation Kit (Vazyme Biotech, Inc, Nanjing, China) according to the manufacturer’s instructions. The quantity and quality of total RNA were assessed using 1% agarose gels and a Nanodrop ND 2000 Spectrophotometer. The integrity and concentration of total RNA were assessed using a Bioanalyzer 2100 RNA 6000 Nano Kit (Agilent Technologies, Santa Clara, CA, USA).

The poly(A) mRNA was isolated from total RNA through an Oligotex mRNA Mini Kit (Qiagen, Inc., Valencia, CA, USA). After construction and normalization, nine cDNA libraries were sequenced on the Illumina Hiseq 6000 Sequencing platform (Illumina, Inc., San Diego, CA, USA) and 150 bp paired-end reads were generated. The raw data were processed by removing the low-quality sequences (reads with more than 50% Q < 19 bases), the adaptor-pollute sequences, and sequences with ambiguous base reads accounting for more than 5%.

To understand their functions, the obtained clean reads were aligned to the *R. rugosa* genome database (Chen et al., 2021) by HISAT2 v2.0.5. New transcript prediction was performed *via* StringTie 1.1.3b (Pertea et al., 2015). The expression levels of genes were calculated as Fragments Per Kilobase of exon model per Million mapped fragments (FPKM), which eliminates the effect of sequencing depth and gene length on gene expression levels and permits direct data comparisons by the DESeq method. The differentially expressed genes (DEGs) between different germplasms were identified if their log_2_|Fold Change| was over 2 with a *p*-value < 0.05. And then the GO and KEGG enrichment according to the identified DEGs were analyzed. The key differentially expressed structural genes and TFs involved in anthocyanin biosynthesis were identified and their expression patterns were analyzed. The correlation analysis between different factors was performed *via* the online platform Metware Cloud (https://cloud.metware.cn). Person correlation coefficient was used to screen the correlation between anthocyanins and genes, and between structural genes and TFs, with correlation coefficients > 0.8 and *p*-value< 0.05 as the selection criteria.

### qRT-PCR analysis

Quantitative real-time PCR (qRT-PCR) were carried out for verifying the transcriptome data and analyzing the expression patterns of key structural genes and TFs related to anthocyanin biosynthesis in the petals of six *R. rugosa* germplasms. All actions were done on a CFX96 Real-time System using SYBR Green Dye according to previous study (Wu et al., 2020). The expression patterns were analyzed through the 2^-ΔΔCt^ method with *RrGADPH* as an internal control (Sui et al., 2019). All primers used in this study are listed (**Supplementary Table 2**). There were three biological replicates for each sample.

## Results

### Anthocyanin type and content analyses

**Figure 1:**
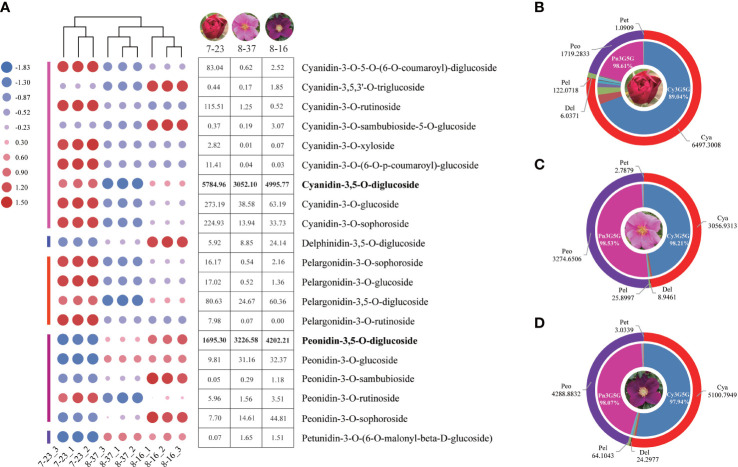
Anthocyanin type and content analyses in three *R. rugosa* germplasms. **(A)** Change trends of 20 main anthocyanins in 7-23, 8-37, and 8-16. **(B)** Anthocyanin distribution in petals of 7-23. **(C)** Anthocyanin distribution in petals of 8-37. **(D)** Anthocyanin distribution in petals of 8-16.

A total of 37 anthocyanins and 11 other flavonoids in 108 anthocyanin databases were identified from the petals of three *R. rugosa* germplasms. All the 37 anthocyanins were present in 7-23, of which 33 and 32 types were detected in 8-37 and 8-16, respectively. The anthocyanin concentrations ranged from 0 to 5863.44 ug g^-1^, and there were 17 trace anthocyanins with the content lower than 1ug g^-1^ in all the three germplasms (**Supplementary Table 1**). The 20 main anthocyanins with the content higher than 1ug g^-1^ were clustered into five classes, such as cyanidins (9), peonidins (5), pelargonidins (4), delphinidins (1) and petunidins (1). This showed that there were three shunts responsible for anthocyanin synthesis in each *R. rugosa* germplasm, namely cyanidin, pelargonidin, and delphinidin biosynthesis pathway. However, the accumulation patterns of anthocyanins in the three *R. rugosa* germplasms were significantly different (**Figure 1A**).

In the three germplasms, the sum of cyanidins and peonidins accounted for 98.45% (7-23), 99.41% (8-37), and 99.04% (8-16) of the total detected anthocyanin, respectively (**Figures 1B** **–D**), indicating that cyanidins and peonidins were the major anthocyanin types in the petals of tested germplasms, and cyanidin biosynthesis shunt may be the key pathway for flower color formation in *R. rugosa*. Moreover, the Cy3G5G accounted for 89.04% (7-23), 98.21% (8-37), and 97.94% (8-16) of the total cyanidin content, while the Pn3G5G accounting for 98.61% (7-23), 98.53% (8-37), and 98.07% (8-16) of the total peonidins content, respectively. The ratio of Cy3G5G to Pn3G5G in 7-23 (3.41) was significantly higher than these in 8-37 (0.93) and 8-16 (1.19), which suggested that the ratio of Cy3G5G to Pn3G5G may play an important role in the color formation of *R. rugosa* petals.

It should be noted that four kinds of delphinidins, such as delphinidin-3-*O*-(6-*O*-acetyl)-glucoside, delphinidin-3-*O*-(6-*O*-*p*-coumaroyl)-glucoside, delphinidin-3-*O*-rutinoside-5-*O*-glucoside, and delphinidin-3,5-*O*-diglucoside (Dp3G5G), were identified from the petals of three *R. rugosa* germplasms (**Supplementary Table 1**). Among them, the contents of Dp3G5G were highest, i.e., 5.92 ug g^-1^ in 7-23, 8.85 ug g^-1^ in 8-37, and 24.14 ug g^-1^ in 8-16 (**Figure 1A**). Obviously, the content of Dp3G5G in purple-red-flowered germplasm with somewhat unique and visible blue hue (8-16) was most abundant, which was 4.08- and 2.73-fold of red-flowered (7-23) and pink-flowered (8-37) germplasms, respectively.

### Quantitative analysis of Cy3G5Gand Pn3G5G

**Figure 2:**
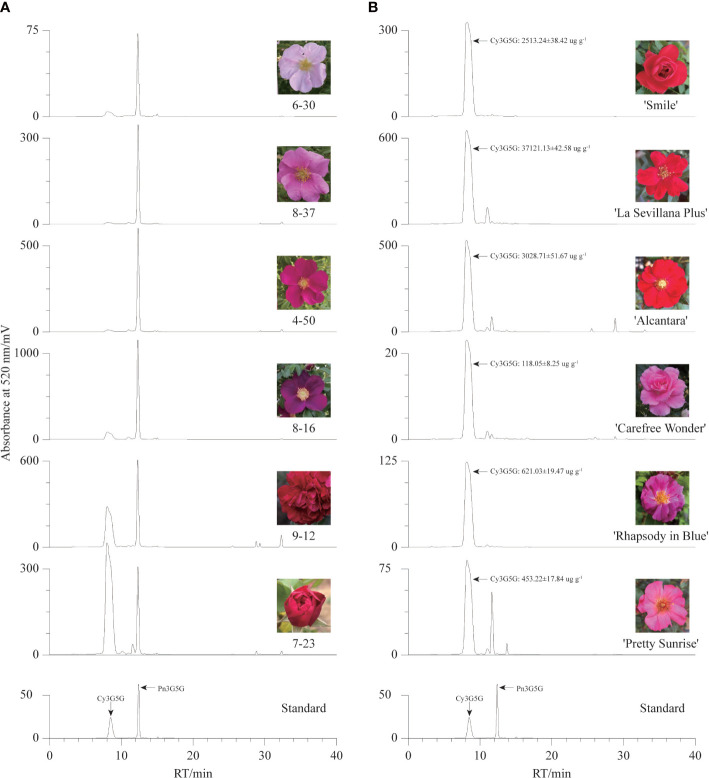
Anthocyanin HPLC chromatographic profiles of different germplasms. **(A)** The chromatogram maps of six *R. rugosa* germplasms. **(B)** The chromatogram maps of six *R. hybrida* cultivars.

To confirm our hypothesis, the contents of Cy3G5G and Pn3G5G were quantitatively analyzed in the petals of six *R. rugosa* germplasms with representative color by HPLC. There were two dominant chromatographic peaks, Cy3G5G and Pn3G5G, were found in the anthocyanin HPLC chromatographic profiles of six *R. rugosa* petals (**Figure 2A**). The petals of different germplasms gradually darkened in color (from pink to purple/red) with the increase of the sum content of Cy3G5G and Pn3G5G (**Table 1**). What’s more, the ratio of Cy3G5G to Pn3G5G (0.76~1.92) in the petals of red-flowered *R. rugosa* germplasm (9-12 and 7-23) was much higher than that in other flowered germplasms (0.03~0.13). It was verified that Cy3G5G and Pn3G5G were the key anthocyanins for the color formation in *R. rugosa* petals, of which sum content were related to the intensity of petal color, such as pink or purple, light- or dark-red. And then, the ratio of the two anthocyanins played important roles in the petal color modification in *R. rugosa*, especially high relative content of Cy3G5G may be a key factor for the red color formation. However, the relationship between absolute content of Cy3G5G and red petal presence needed further study.

**Table 1:** Contents of Cy3G5G and Pn3G5G in six *R. rugosa* germplasms based on HPLC analysis.

Germplasms	Cy3G5G content/ug g^-1^	Pn3G5G content/ug g^-1^	Sum content/ug g^-1^	Cy3G5G/Pn3G5G
6-30	25.59 ± 1.73	259.59 ± 27.65	285.18 ± 29.14	0.10
8-37	37.48 ± 2.69	1267.69 ± 52.07	1305.17 ± 54.72	0.03
4-50	57.80 ± 3.61	1622.93 ± 67.02	1680.73 ± 63.69	0.04
8-16	403.60 ± 15.42	3209.37 ± 65.92	3612.97 ± 81.26	0.13
9-12	1614.69 ± 66.49	2120.37 ± 83.30	3735.06 ± 17.49	0.76
7-23	2063.79 ± 55.34	1072.61 ± 46.37	3336.40 ± 13.57	1.92

And then, six *R. hybrida* cultivars including *R. hybrida* ‘Smile’, the male parent and contributors to the red-petal characteristics of 7-23 and 9-12, were used to measure the Cy3G5G content in the petals. In the petals of all six *R. hybrida* cultivars, Cy3G5G was the major anthocyanin with high quantitative concentration (**Figure 2B**). The contents of Cy3G5G in red-flowered *R. hybrida* cultivars (2513.24 ug g^-1^~3714.13 ug g^-1^) were much higher than that in the pink- or purple-flowered *R. hybrida* cultivars (119.18 ug g^-1^~626.16 ug g^-1^). Therefore, the ratio of Cy3G5G to Pn3G5G can affect the petal color hue of *R. rugosa*, that is, red or pink/purple. Meanwhile, it might be necessary to maintain both high relative and high absolute content of Cy3G5G for forming red-colored petals in *R. rugosa* and *R. hybrida*.

### Global analysis of RNA-seq data

To further explore the key genes associated with petal color formation, nine cDNA libraries were constructed and the raw data was deposited at the NCBI Sequence Read Archive (SRA) under accession numbers SRR20883375~SRR20883383. An average 49869442 (97.29%) of clean reads was obtained after removing the adaptor and low-quality reads (**Supplementary Table 3**), and the percentages of Q20, Q30 base rates were more than 96.78% and 91.35%, respectively. The average GC content was 45.90% and the rate of total mapping ranged from 74.78% to 85.22%.

### GO and KEGG analyses of DEGs

**Figure 3:**
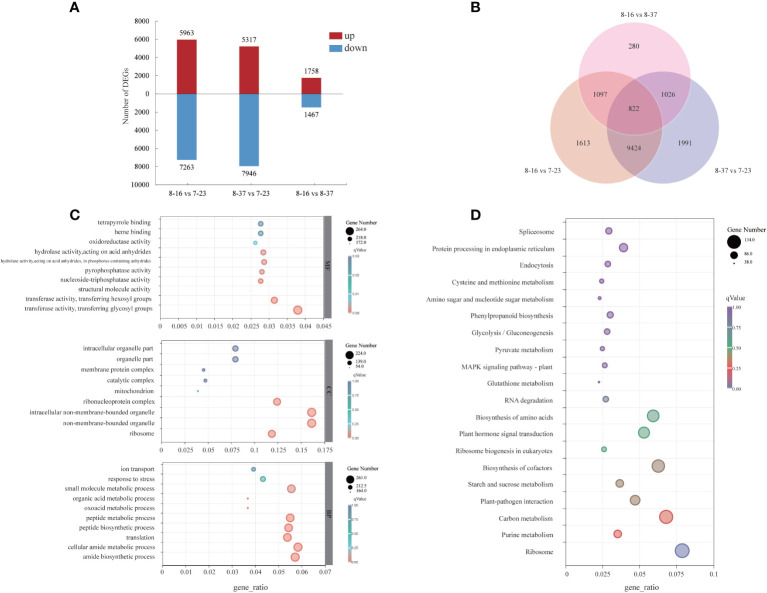
Distribution and annotation analyses of DEGs among three *R. rugosa* germplasms. **(A)** The number of up-regulated and down-regulated genes. **(B)** Venn diagram depicting the shared and specific DEGs among three compare combinations. **(C)** GO classifications of DEGs. **(D)** KEGG functional analysis of DEGs.

To investigate the dynamic expression patterns of the specific genes associated with anthocyanin accumulation, the transcriptome profiles of different germplasms were compared. The DEGs between different germplasms were identified with log_2_|Fold Change| ≥2 and *p*-value < 0.05. A total of 12956 DEGs had different expression patterns between 8-16 and 7-23 with 5693 up-regulated genes and 7263 down-regulated genes (**Figure 3A**). Taking 7-23 as control, a total of 13263 DEGs were expressed differentially in 8-37, with more down-regulated genes (7946) than up-regulated genes (5317). There were 3225 DEGs which had different expression patterns between 8-16 and 8-37, including 1758 up-regulated genes and 1467 down-regulated genes. 16253 genes were found in three compare combinations, of which 822 DEGs were present in all combinations (**Figure 3B**).

For the GO annotation, the identified DEGs were assigned into three main GO functional categories (**Figure 3C**), including biological process (BP), cellular component (CC), and molecular function (MF). For the BP category, the three most abundant sub-categories were “cellular amide metabolic process” (261 DEGs), “amide biosynthetic process” (256 DEGs), and “small molecule metabolic process” (248 DEGs). The majority of DEGs of CC category were assigned into “non-membrane-bounded organelle” (224 DEGs), “intracellular non-membrane-bounded organelle” (224 DEGs), and “ribonucleoprotein complex” (172 DEGs). The three most abundant sub-categories which the MF category was divided were “transferase activity, transferring glycosyl groups” (264 DEGs), “transferase activity, transferring hexosyl groups” (219 DEGs), and “hydrolase activity, acting on acid anhydrides” (199 DEGs). In addition, 12 DGEs in MF category were involved in “*O*-methyltransferase activity”.

A total of 1706 DEGs were assigned into 112 KEGG pathways (**Figure 3D**) with three most abundant pathways as “Ribosome” (134 DEGs), “Carbon metabolism” (116 DEGs), and “Biosynthesis of cofactors” (107 DEGs). In addition, some DEGs were mapped to the pathways related to anthocyanin biosynthesis, such as “Phenylpropanoid biosynthesis” (ath00940, 51 DEGs), “Phenylalanine metabolism” (ath00360, 8 DEGs), “Flavonoid biosynthesis” (ath00941, 9 DEGs).

### Identification of DEGs related to anthocyanin biosynthesis

All structural genes from DEGs involved in anthocyanin biosynthesis were identified (**Supplementary Table 4**). There were 32 DEGs encoding 10 enzymes, such as PAL, CHS, F3H, F3’H, ANS, leucoanthocyanidin reductase (LAR), anthocyanidin 3-*O*-glucosyltransferase (BZ1), anthocyanidin 3-*O*-glucoside 5-*O*-glucosyltransferase (UGT75C1), GT1, and anthocyanin *O*-methyltransferase (AOMT), found from the compare combination of 8-37 vs 7-23. A total of 33 DEGs were identified to encode 13 enzymes including PAL, 4CL, CHS, F3H, FLS, F3’H, F3’5’H, ANS, LAR, BZ1, UGT75C1, GT1, and AOMT in the compare combination of 8-16 vs 7-23. In the compare combination of 8-16 vs 8-37, 10 DEGs encoding 5 enzymes (CHS, FLS, BZ1, UGT75C1, and GT1) were identified. As a total, 36 structural genes from DEGs encoding 13 enzymes were analyzed in three *R. rugosa* germplasms.

MYB, WD40, and bHLH were the major TFs associated with the regulation of anthocyanin biosynthesis. A total of 55 *MYB*s, 25 *WD40*s, and 64 *bHLH*s were identified as the DEGs in the compare combination of 8-37 vs 7-23 (**Supplementary Table 5**). There were 56 *MYB*s, 24 *WD40*s, and 62 *bHLH*s found as the DEGs in the compare combination of 8-16 vs 7-23. For the compare combination of 8-16 vs 8-37, 14 *MYB*s, 4 *WD40*s, and 5 *bHLH*s were identified. Overall, there were 173 differentially expressed TFs found among three *R. rugosa* germplasms, including 70 *MYB*s, 29 *WD40*s, and 74 *bHLH*s, respectively.

### The analysis of qRT-PCR verification

To verify the reliability of the RNA-seq data, 16 DEGs were subjected to qRT-PCR. The expression patterns of 14 DEGs were significantly correlated with the results of RNA-seq (R > 0.80). The result showed that the gene expression profiles were well consistent with the RNA-seq data, which demonstrated the credibility of the data generated in our study (**Supplementary Figure 1**).

### The analysis of key structural genes related to color formation

**Figure 4:**
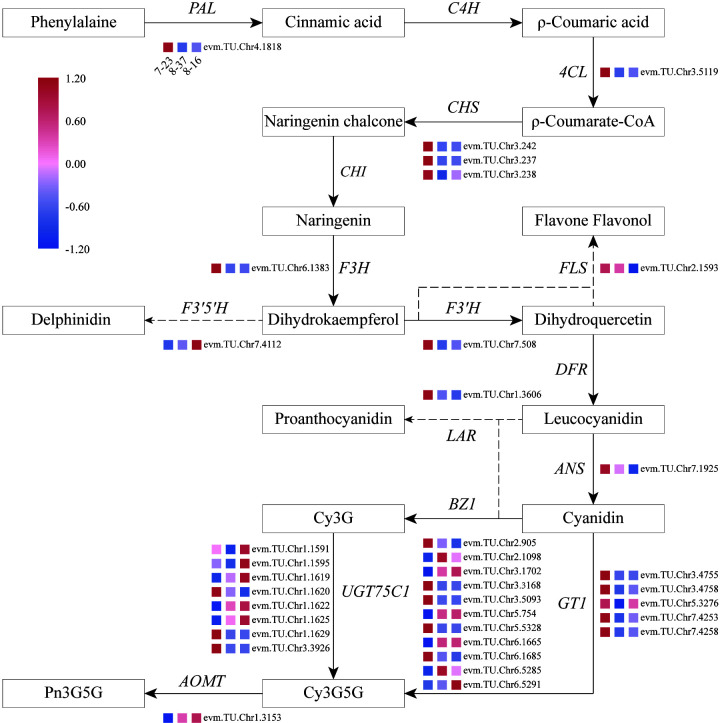
Overview of the pathway of anthocyanin biosynthesis and temporal expressional patterns of related DEGs. *4CL*, 4-coumaroyl-CoA synthase; *ANS*, anthocyanidin reductase; *AOMT*, anthocyanin *O*-methyltransferase; *BZ1*, anthocyanidin 3-*O*-glucosyltransferase; *C4H*, Cinnamic acid 4-hydroxylase; *CHI*, Chalcone isomerase; *CHS*, Chalcone synthase; *DFR*, Dihydroflavonol 4-reductase; *F3H*, Flavanone-3-hydroxylase; *F3’5’H*, Flavonoid-3’5’-hydroxylase; *F3’H*, Flavonoid 3’-hydroxylase; *FLS*, Flavonol synthase; *GT1*, anthocyanidin 5,3-*O*-glucosyltransferase; *LAR*, leucoanthocyanidin reductase; *PAL*, Phenylalanine ammonia-lyase; *UGT75C1*, anthocyanidin 3-*O*-glucoside 5-*O*-glucosyltransferase.

Focusing on key enzymes involved in anthocyanin biosynthesis associated with petal color formation, a summary overview of anthocyanin accumulation processes in *R. rugosa* petals was constructed (**Figure 4**). Compared to 8-16 and 8-37, 7-23 presented 35 DEGs in the upstream pathway of cyanidin biosynthesis with 11 down-regulated genes encoding F3’5’H (1), BZ1(6), and UGT75C1(4), and 24 up-regulated genes encoding PAL (1), 4CL (1), CHS (3), F3H (1), FLS (1), F3’H (1), ANS (1), LAR (1), BZ1(5), UGT75C1(4), and GT1(5). Among the 24 DEGs, the genes with log_2_|Fold Change|≥4 accounted for more than 66%. In the previous result, we found that the sum content of Cy3G5G and Pn3G5G in 7-23 was approximately consistent with those of the other two germplasms with the ratios of 1.20 (8-37) and 0.81 (8-16), respectively. Meanwhile, the content of procyanidin in 7-23 was much higher than these in the other two germplasms with the ratios of 11.78 (8-37) and 27.03 (8-16) (**Table 2**). Between 8-37 and 8-16, there were ten DEGs in the upstream pathway of cyanidin biosynthesis with seven up-regulated genes encoding CHS (1), BZ1(2), UGT75C1(3), and GT1(1), and three down-regulated genes encoding FLS (1) and BZ1 (2). The coordinate expression of ten DEGs identified between 8-37 and 8-16 promoted the biosynthesis of Cy3G5G and Pn3G5G and increased the sum content in purple-red-colored petals (8-16). Therefore, in the upstream pathway of cyanidin biosynthesis, the key genes encoding 12 enzymes co-expressed to regulate the amount of Cy3G5G and Pn3G5G in *R. rugosa* petals and then determined the color intensity of petals, namely pinkish or purplish. It is worth mentioning that the expression level of *RrF3’5’H* (evm.TU.Chr7.4112) in purple-red-flowered 8-16 with somewhat unique and visible blue hue was 4.20- and 3.04-fold of red-flowered 7-23 and pink-flowered 8-37, which showed the similar trend as the content of Dp3G5G.

**Table 2:** FPKM values (F) of three DEGs and contents (C) of related metabolite.

Germplasms	F*_LAR_*	F*_F3’5’H_*	F*_AOMT_*	C_Pr_/ug g^-1^	C_Cy3G5G+Pn3G5G_/ug g^-1^	C_Pn3G5G_/C_Cy3G5G +Pn3G5G_
7-23	53.45 ± 2.07	0.92 ± 0.37	0.34 ± 0.08	138.63 ± 7.94	7480.27 ± 168.96	22.66%
8-37	2.39 ± 0.15	1.27 ± 0.31	11.04 ± 0.66	11.77 ± 0.65	6228.68 ± 14.19	51.80%
8-16	1.26 ± 0.65	3.86 ± 0.18	21.76 ± 4.55	5.13 ± 0.13	9201.98 ± 187.53	45.71%

LAR, leucoanthocyanidin reductase; F3’5’H, flavonoid-3’5’-hydroxylase; AOMT, anthocyanin O-methyltransferase; Pr, proanthocyanidin; Cy3G5G, cyanidin 3,5-O-diglucoside; Pn3G5G, peonidin 3,5-O-diglucoside.

In the downstream pathway of cyanidin biosynthesis, only one structural gene, *RrAOMT* (evm.TU.Chr1.3153), expressed and catalyzed Cy3G5G to form Pn3G5G. The expression level of *RrAOMT* in 7-23 accounted for 3.07% and 1.56% of these in 8-37 and 8-16, respectively. The high expression level of *RrAOMT* in 8-37 and 8-16 promoted 51.80% and 45.71% Cy3G5G to transform Pn3G5G, making the petals pinkish or purplish. Conversely, the low expression of *RrAOMT* in 7-23 with red-flowered petals promoted only 22.66% Cy3G5G to form Pn3G5G (**Table 2**). Overall, the *RrAOMT* presented in the downstream pathway of cyanidin biosynthesis was an extremely important key gene to regulate the petal color during flowering. By regulating the ratio of Cy3G5G to Pn3G5G in the petals, *RrAOMT* determined the color hue of petals in *R. rugosa*, namely red and pink/purple.

In general, there were three anthocyanin transportation models, including GST (Glutathione S-Transferase), MRP/ABCC (Multidrug resistance-associated protein), and MATE (Multidrug and toxic compound extrusion). A total of eight, three, and three DEGs were identified to be involved in GST, MRP/ABCC, and MATE, respectively (**Supplementary Table 6**). Five and six GSTs showed up-regulated in 7-23 germplasm with red petals compared to 8-16 and 8-37, respectively. All the DEGs of MRP/ABCC showed peak expressions in 8-16, while the expressions of one and two MATEs peaked in 7-23 and 8-37, respectively.

### The analysis of key TFs related to color formation

**Figure 5:**
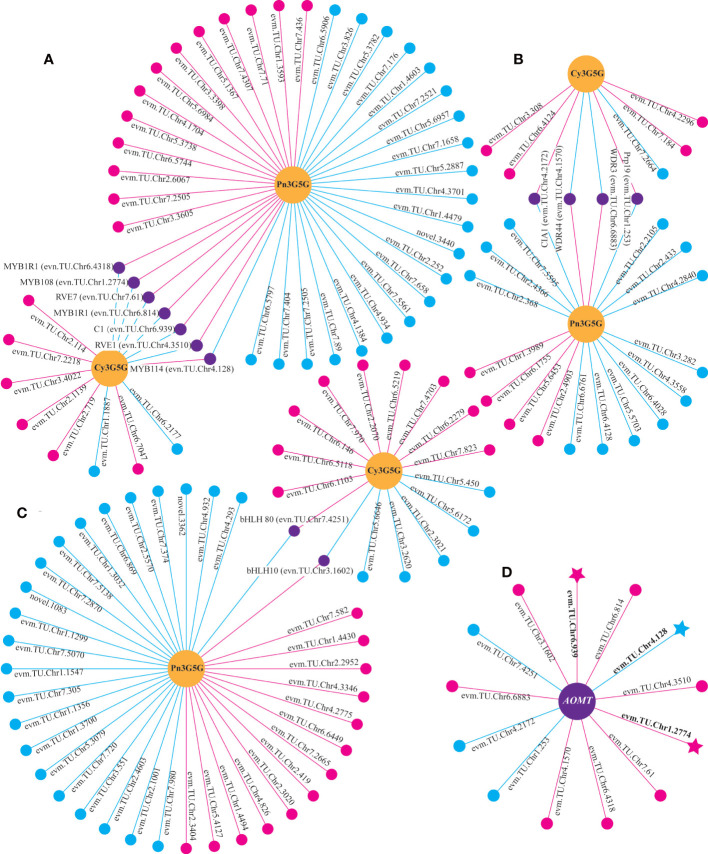
Correlation analysis between differentially expressed genes and metabolites. **(A)** Correlation analysis of between *MYB*s and two metabolites. **(B)** Correlation analysis of between *WD40*s and two metabolites. **(C)** Correlation analysis of between *bHLH*s and two metabolites. **(D)** Correlation analysis of between TFs and *AOMT*. Circles colored in “yellow” mean metabolites. Circles colored in “pink”, “blue”, and “purple” mean structural genes or TFs. Lines colored in “pink” and “blue” represent positive and negative correlations, respectively.

Through the correlation analysis between differentially expressed TFs and the content of Cy3G5G and Pn3G5G, it was found that 13 TFs (seven for *MYB*s, four for *WD40*s, and two for *bHLH*s) were significantly correlated with both Cy3G5G and Pn3G5G contents, respectively, with opposite correlation (**Figures 5A** **–C**). It indicated that these 13 TFs may play important roles in regulating Cy3G5G and Pn3G5G contents. We got the similar results by analyzing the correlation between the TFs and the contents of cyanidins and peonidins. The correlation analysis showed significant correlation between all the 13 TFs and *RrAOMT* (**Figure 5D**), indicating that these 13 TFs (TFs group A) determined the *R. rugosa* petal color formation by controlling the expression of *RrAOMT* and then regulating the ratio of Cy3G5G to Pn3G5G.

**Figure 6:**
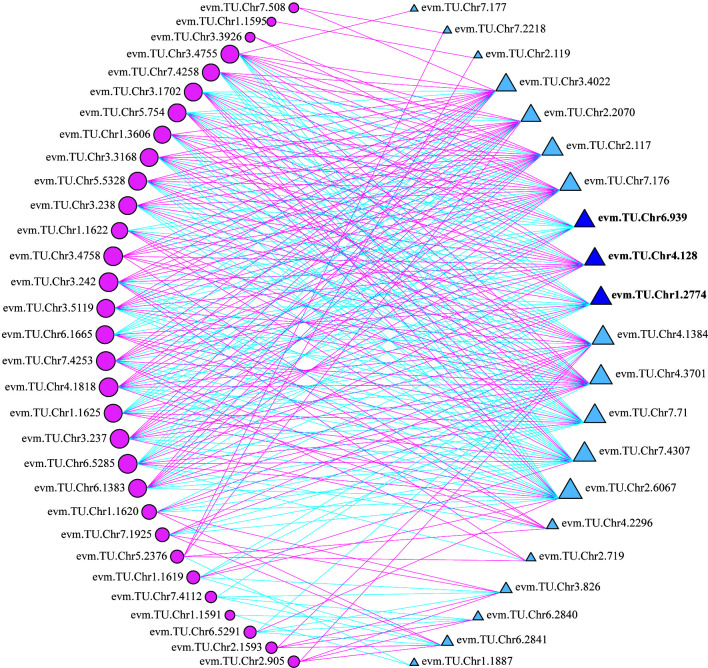
Correlation analysis of between differentially expressed TFs and structural genes. Circles colored in “pink” and triangles colored in “blue” mean structural genes and TFs respectively. Lines colored in “red” and “blue” represent positive and negative correlations, respectively.

According to the transcriptome annotation and blast results, 22 TFs related to anthocyanin biosynthesis were selected to analyze the correlation with 35 structural genes form the upstream pathway of cyanidin biosynthesis (**Supplementary Table 7**). It indicated that 21 TFs had significant correlation with multiple structural genes, of which 12 TFs were correlated with more than 19 genes (**Figure 6**). The 12 TFs (TFs group B) may play important roles in regulating the total anthocyanin content in the upstream pathway of cyanidin biosynthesis.

Moreover, three TFs, *RrMYB108* (evm.TU.Chr1.2774), *RrC1* (evm.TU.Chr6.939), and *RrMYB114* (evm.TU.Chr4.128) were identified from both TFs group A and B. Sequence alignment analysis showed that protein sequence similarity between three TFs and the homologous sequences involved in anthocyanin biosynthesis from other species ranged from 31.56% to 66.20% (**Supplementary Figure 2**). The three TFs may be the key factors determining *R. rugosa* petal color with multiple functions, not only promoting the total anthocyanin biosynthesis by regulating structural genes in the upstream pathway, but also adjusting the ratio of Cy3G5G to Pn3G5G *via* controlling the *RrAOMT* expression in the downstream pathway of anthocyanin biosynthesis. Further analysis revealed that the three TFs were significantly associated with the same genes. *RrMYB114* was positively correlated with 13 of 19 structural genes from the upstream pathway of cyanidin biosynthesis and negatively correlated with *RrAOMT*, the structural genes from the downstream pathway. It indicated that *RrMYB114* may play a key role in the positive regulation of the total anthocyanin content and the ratio of Cy3G5G to Pn3G5G. The correlation of *RrMYB108* and *RrC1* was opposite with each structural gene involved in anthocyanin biosynthesis (**Supplementary Table 8**).

### The expression patterns of four key genes in six *R. rugosa* germplasms with different petal color

**Figure 7:**
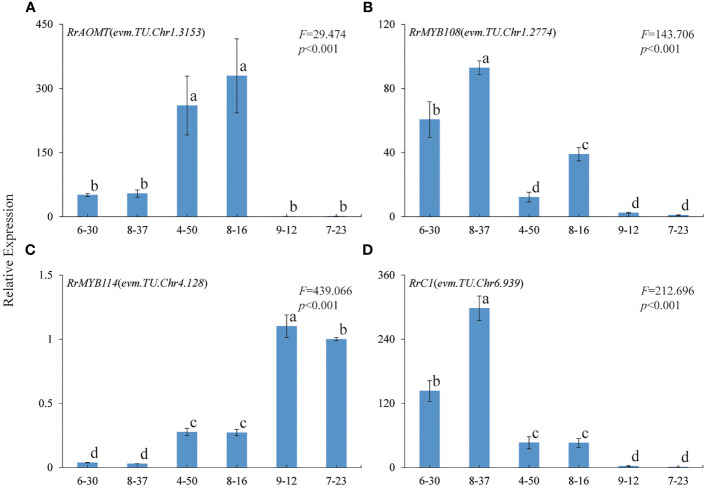
Expression patterns of four key genes in six R. rugosa germplasms. **(A)** RrAOMT. **(B)** RrMYB108. **(C)** RrMYB114. **(D)** RrC1. The different small letters indicate significant difference between germplasms at α=0.05.

In order to further verify the relationship between the four genes and *R. rugosa* petal color formation, the expression trends of *RrAOMT* (evm.TU.Chr1.3153), *RrMYB108* (evm.TU.Chr1.2774), *MYB114* (evm.TU.Chr4.128),and *RrC1* (evm.TU.Chr6.939) in six *R. rugosa* germplasms were measured (7-23 as control). The expression trends of four key genes in six *R. rugosa* germplasms were different with the change of flower color. The expression levels of *RrAOMT* in red-flowered germplasms were extremely low, but that in pink- and purple-flowered germplasms were high and increased gradually with the flower color deepening (**Figure 7A**). The expression levels of *RrMYB114* in red-flowered germplasms were extremely high, but that in pink- and purple-flowered germplasms were low and increased gradually with the flower color deepening. That is to say, the expression levels of *RrMYB114* and *RrAOMT* shared a similar trend in pink- and purple-flowered germplasms, but a contrary trend in red-flowered germplasms (**Figure 7C**). The expression levels of *RrMYB108* and *RrC1* in red-flowered germplasms were extremely low, but that in pink- and purple-flowered germplasms were high and reduced gradually with the flower color deepening. In other words, the expression trend of *RrMYB108* and *RrC1* in pink- and purple-flowered germplasms were contrary to *RrAOMT* and *RrMYB114*, but in red-flowered germplasms were consistent with *RrAOMT* and contrary to *RrMYB114* (**Figures 7B, D**).

## Discussion

### Anthocyanin identification and coloration regular of *R. rugosa* flowers

The monotonous flower color is an important limiting factor for the landscape application of *R. rugosa*. Anthocyanin is the main coloration compound in the petals of *R. rugosa*. Previous investigates showed that there was almost no anthocyanin in the petals of *R. rugosa* ‘Bai Zizhi’, and peonidins were the main pigment compositions in the petals of *R. rugosa* ‘Zi zhi’ and *R. rugosa* ‘Fen Zizhi’ (Zhang et al., 2015; Sheng et al., 2018). In this study, we found that the sum of cyanidins and peonidins accounted for more than 98% of the total detected anthocyanin content, which indicated cyanidin biosynthesis shunt was the dominant pathway for anthocyanin accumulation in *R. rugosa*, and peonidins showed obvious content advantage compared with cyanidins in most *R. rugosa* germplasms. These were the key reasons why the petal color of *R. rugosa* was monotonous and mainly pink/purple. Furthermore, we found that Cy3G5G and Pn3G5G were the primary anthocyanins, and the sum content of Cy3G5G and Pn3G5G was responsible for the flower color intensity of *R. rugosa* (**Figures 1**, **2** and **Table 1**). These results were consistent with the conclusions derived from Zhang et al. (2015) and Sheng et al. (2018), and provided further supplement and deepening of *R. rugosa* flower color formation mechanism.

Red has always been loved by the Chinese people because of its strong visual attraction and celebratory meaning, which brings high market value to red-flowered cultivars. Unfortunately, there were many pink and purple-red cultivars without red cultivars in *R. rugosa* (Sui et al., 2018). We obtained two red-flowered *R. rugosa* germplasm, namely 7-23 (bright red) and 9-12 (dark red with somewhat purple) derived from interspecific hybridization between *R. rugosa* and *R. hybrida*, which could be two important materials for further study on the mechanism of flower color formation in *R. rugosa*. For plants with anthocyanin as the essential coloration compound of petals, pelargonidins and cyanidins were confirmed as the main contributors to the red petal phenotype (Du et al., 2015; Du et al., 2017; Zhang et al., 2018; Meanchainiboon et al., 2020). However, the petals of many plants with pelargonidins or cyanidins as dominant anthocyanins showed pink color (Jiao et al., 2020; Fu et al., 2021), which may be related to the low absolute content of pelargonidins or cyanidins. After maize *Lc* was transferred into tobacco (*Nicotiana tobacum*), the cyanidin content of tobacco petals increased by 38-fold, and the petal color changed from light pink to dark red (Huang et al., 2016), which might be main evidence of the above inference. In this study, quantitative analysis of Cy3G5G and Pn3G5G in petals of six *R. rugosa* germplasms and six *R. hybrida* cultivars showed that high relative content and absolute content of Cy3G5G were two essential conditions for red petals of *R. rugosa* and *R. hybrida* (**Table 1**, **Figure 2**). The result not only further revealed the coloration regular of *R. rugosa* petals but also provided new evidence for the coloration mechanism of plant red petals.

A large amount of delphinidin-based anthocyanins accumulation was the primary condition to form blue petals (Yoshida et al., 2009; Noda et al., 2017). *R. hybrida* lacks blue flower cultivars due to the absence of delphinidin-based anthocyanins (Katsumoto et al., 2007). Moreover, delphinidins were not detected in other *Rosa* species (Mikanagi et al., 2000). However, delphinidins were identified from the petals of *R. rugosa* ‘Zizhi’, and this is the first time for delphinidins found in *Rosa* species (Zhang et al., 2015). In this study, Dp3G5G was detected again in the petals of three other *R. rugosa* germplasms (7-23, 8-37, and 8-16) (**Figure 1**), meanwhile *F3’5’H* (evm.TU.Chr7.4112), encoding a key enzyme related to delphinidin biosynthesis, was also identified (**Figure 4**). Furthermore, the petals of 8-16 germplasm were deep purple-red color with somewhat unique and visible blue hue, and the content of Dp3G5G and the expression level of *RrF3’5’H* in which were significantly higher than the other two red- and pink-flowered germplasms. These results indicated that *R. rugosa* possessed a delphinidins metabolic pathway, which could provide a new gene resource for flower color improvement in the genus *Rosa*.

### Key candidate structural genes responsible for anthocyanin synthesis in *R. rugosa* flowers

Anthocyanin synthesis pathway was divided into three branches, which synthesized cyanidins (methylated to form paeonidins), pelargonidins and delphinidins (methylated to form petunidins and malvidins), respectively (Jaakola, 2013). The biosynthesis of these anthocyanins is co-regulated by a series of structural genes, such as *CHS*, *CHI*, *F3H*, *F3’H*, *F3’5’H*, *DFR*, *ANS*, and *GT* (Koes et al., 2005; Jaakola, 2013; Iaria et al., 2016; Goswami et al., 2018). In *R. rugosa*, the information of structural genes involved in anthocyanin biosynthesis was extreme sparse. Up to now, only two genes, *RrGT1* and *RrGT2*, had been identified and confirmed to participate in anthocyanin biosynthesis of *R. rugosa* (Sui et al., 2018; Sui et al., 2019). In this study, we found that the cyanidins synthesis pathway was the most dominant branch in three anthocyanin biosynthesis pathways of *R. rugosa* flowers. The expression of three transcripts annotated as *F3’H* was higher than that of *F3’5’H* from delphinidin branch, consistent with the metabolite profile analysis that the sum contents of cyanidin and peonidin was much greater than the sum content of delphinidin and petunidin in 7-23, 8-37, and 8-16, respectively. Otherwise, the inhibition of pelargonidins and delphinidins branches may be related to the substrate specificity of *DFR*, a structural gene involved in the upstream pathway of anthocyanin biosynthesis (Davies et al., 2003).

In this study, we found that 35 DEGs encoding 12 key enzymes in the upstream pathway of cyanidin biosynthesis co-expressed to regulate the sum content of Cy3G5G and Pn3G5G in *R. rugosa* petals and determine the intensity of petals color, namely pink or purple (**Figure 4**). Interestingly, 24 DEGs in 7-23 were significantly up-regulated compared with 8-37 and 8-16, but the sum content of Cy3G5G and Pn3G5G in 7-23 was only 1.20- and 0.81-fold that in 8-37 and 8-16, meanwhile the content of procyanidins was 11.78- and 27.03-fold that in 8-37 and 8-16 (**Table 2**). Accordingly, we speculated that the high expression level of *RrLAR* (evm.TU.Chr1.3606) in 7-23, which was 22.36- and 42.42-fold of 8-37 and 8-16 expression levels, reduced the production of Cy3G5G and Pn3G5G by catalyzing a large amount of leucocyanidins to form procyanidins. Otherwise, the transportation model of GST showed signific expression in the red flowered germplasm (7-23), which indicated that the GST may be the main anthocyanin transportation during the red flower color formation.

It was worth noting that there was only one DEG (evm.TU.Chr1.3153) encoding AOMT, a key enzyme from the downstream pathway of cyanidin synthesis. As we all know, AOMT play an important role in anthocyanin methylation. It can increase diversity and improve the stability of anthocyanin (Roldan et al., 2014; Du et al., 2015). Few *AOMTs* were identified and characterized such as *CkmOMT2* in cyclamen, *VvAOMT* in grape, *AnthOMT* in tomato, *PsAOMT* in purple-flowered tree peony, and *PtAOMT* in red-flowered herbaceous peony, respectively (Kondo et al., 2009; Hugueney et al., 2009; Akita et al., 2011; Roldan et al., 2014; Du et al., 2015). These *AOMTs* diversified the colors of flowers and fruits by regulating the methylation of cyanidins and delphinidins. Among them, only *PsAOMT* and *PtAOMT* were responsible for the methylation of cyanidins and varied the color of cyanidin-based anthocyanins from red to purple (Du et al., 2015). In the present study, a large amount of cyanidins were methylated to form peonidins under high-expression of *RrAOMT* in the petals of pink-flowered (8-37) and purple-flowered (8-16) germplasms, and only a small amount of cyanidins were methylated to form peonidins under low-expression of *RrAOMT* in the petals of red-flowered (7-23) germplasm (**Table 2**). Furthermore, we measured the expression levels of *RrAOMT* in six *R. rugosa* germplasm with different petals color. The result showed that the expression levels of *RrAOMT* in red-flowered germplasms were extremely low, but that in pink- and purple-flowered germplasms were high and increased gradually with the flower color deepening (**Figure 7A**). These indicated that *RrAOMT* was a critical structural gene, which was responsible for the content ratio of Cy3G5G to Pn3G5G by regulating the methylation of Cy3G5G and determined the color hue of *R. rugosa* petals, namely red or pink/purple. The conclusion was consistent with that of Du et al. (2015). Meanwhile, we also found that high absolute content of Cy3G5G was the other essential condition for red flowers formation of *R. rugosa* besides high relative content (**Table 1** and **Figure 2**). From that, it was presumed that deep purple-red-flowered *R. rugosa* germplasms with high anthocyanin content should be selected as transgenic materials to create new red-flowered *R. rugosa* germplasms by inhibiting *RrAOMT* expression, because light pink-flowered germplasms with low anthocyanin content cannot produce sufficient Cy3G5G by inhibiting *RrAOMT* expression. Taken together, *R. rugosa* can provide a good model system for the investigation of methylation mechanisms of cyanidin-based anthocyanins and their influence on flower coloration.

### Key candidate TFs involved in anthocyanin synthesis in *R. rugosa* flowers

Except to structural genes, MYB, bHLH, and WD40 TFs also play important roles in plant coloration. MYB TFs are generally considered to be the most critical TFs for the synthesis of plant anthocyanins and a large amount of MYB TFs have been characterized (Zhang et al., 2019; Sakai et al., 2019; Wang et al., 2019a; Karppinen et al., 2021; Jiang et al., 2018). Most MYB TFs regulating anthocyanin synthesis in Rosaceae plants were identified from apple, pear, strawberry, and other fruits, such as *MdMYB2* in apple, *PyMYB114* in pear, and *FaMYB10* in strawberry (Yao et al., 2017; Wang et al., 2019b; Jiang et al., 2022). Few MYB TFs regulating anthocyanin synthesis were identified from Rosaceae ornamental plants, such as *RcMYB114* in *Rosa* sp. and *PpMYB108* in peach flower (Li et al., 2022; Khan et al., 2022).

In this study, we identified three R2R3-MYB TFs having conserved domain of R2 and R3, namely *RrMYB108* (evm.TU.Chr1.2774), *RrC1* (evm.TU.Chr6.939), and *RrMYB114* (evm.TU.Chr4.128), by transcriptome annotation, blast, and correlation analysis. The similarity of the three TFs with associated homologous sequences was between 31.56% and 66.20%, indicating that the identified R2R3-MYBs may regulate anthocyanin biosynthesis related to flower color formation in *R. rugosa*. And the expression levels of *RrMYB114* in transcriptome was negatively correlated with the content of Pn3G5G and the expression level of *RrAOMT*, and positively correlated with the content of Cy3G5G and the expression levels of other 13 structural genes, which was just contrary to *RrC1* and *RrMYB108* (**Figures 5**, **6**). Subsequently, we further measured the expression trends of the three TFs in six *R. rugosa* germplasms. The results showed that the expression levels of *RrMYB114* and *RrAOMT* shared a similar trend in four pink- and purple-flowered germplasms, but a contrary trend in two red-flowered germplasms. The expression trend of *RrMYB108* and *RrC1* in four pink- and purple-flowered germplasms were contrary to *RrAOMT* and *RrMYB114*, but in two red-flowered germplasms were consistent with *RrAOMT* and contrary to *RrMYB114* (**Figure 7**). Accordingly, we speculated that these three candidate TFs might play critical roles in the control of petal color in *R. rugosa* by regulating the expression of both *RrAOMT* and other multiple structural genes.

So far, there are a few reports about *MYB108*, *C1*, and *MYB114* involved in the anthocyanin biosynthesis, and they are generally believed to positively regulate anthocyanin synthesis. *PyMYB114* (isolated from Chinese pear) and *PpMYB114* (isolated from ‘Red Zaosu’ pear) were found to interact with other transcription factors to co-regulate fruit anthocyanin biosynthesis in pear (Yao et al., 2017; Ni et al., 2019). Overexpression of *PpMYB108* (isolated from peach flower) was confirmed to significantly increased anthocyanin biosynthesis in tobacco flowers by a positive interaction with *PpDFR* promoter (Khan et al., 2022). *OsC1* (isolated from rice leaves) was verified to activate all anthocyanin biosynthetic genes including *OsCHS*, *OsCHI*, *OsF3’H*, *OsF3H*, *OsDFR* and *OsANS* in rice leaves by forming MYB-bHLH-WD40 complex with *OsRb* and *OsPAC1* (Zheng et al., 2019). In our study, what is inconsistent with the above research results was that the expression levels of *RrMYB108* and *RrC1* were both contrary to the total anthocyanin content in *R. rugosa* petals, which might be induced by functional differentiation of the two TFs in different species. However, the specific functions of *RrMYB108*, *RrC1*, and *RrMYB114* need to be verified by experiments.

## Conclusions

In this study, transcriptome and chemical analyses were used to clarify the flower coloration mechanism in *R. rugosa*. Cy3G5G and Pn3G5G accounted for an extremely high proportion of anthocyanins in petals, while Dp3G5G was present in low amounts but varied in different germplasms. The sum and ratio of Cy3G5G and Pn3G5G contents were responsible for the petal color intensity and hue, respectively. Thirty-five key structural genes involved in the upstream pathway of cyanidin biosynthesis co-expressed to regulate the sum content of Cy3G5G and Pn3G5G, and *RrAOMT* from the downstream pathway regulated the ratio of Cy3G5G to Pn3G5G by methylation. Three candidate TFs, such as *RrMYB108*, *RrC1*, and *RrMYB114*, might regulate the expression of both *RrAOMT* and other multiple structural genes to control the petal colors in *R. rugosa*. This study was the first comprehensive analysis of the flower coloration mechanism in *R. rugosa*, especially in the red-flowered germplasm, and provided a series of candidate genes with applications in the breeding of ornamental plants.

## Data availability statement

The datasets presented in this study can be found in online repositories. The names of the repository/repositories and accession number(s) can be found below: https://www.ncbi.nlm.nih.gov/sra, SRR20883375, SRR20883383.

## Author contributions

YY and QW designed the study. YW, SL, and ZZ performed the experiments and the data analysis. YY, YW, SL, and QW wrote the manuscript. ZX, SQ, and SX participated in the data analysis. All authors contributed to the article and approved the submitted version.

## Funding

This project was funded by the Shandong Agricultural Seeds Engineering Project (2020LZGC011) and the National Science Foundation of China (NSFC) (31870688).

## Acknowledgments

The authors are thankful to Prof. Dekui Zang and Yan Ma for insightful comments on the previous version of this manuscript.

## Conflict of interest

The authors declare that the research was conducted in the absence of any commercial or financial relationships that could be construed as a potential conflict of interest.

## Publisher’s note

All claims expressed in this article are solely those of the authors and do not necessarily represent those of their affiliated organizations, or those of the publisher, the editors and the reviewers. Any product that may be evaluated in this article, or claim that may be made by its manufacturer, is not guaranteed or endorsed by the publisher.
