# Supplementary figures and images for: Transcriptome and chemical analyses revealed the mechanism of flower color formation in Rosa rugosa
Source: Front Plant Sci. 2022 Sep 23;13:1021521. doi: 10.3389/fpls.2022.1021521 (PMC9539313; doi:10.3389/fpls.2022.1021521)

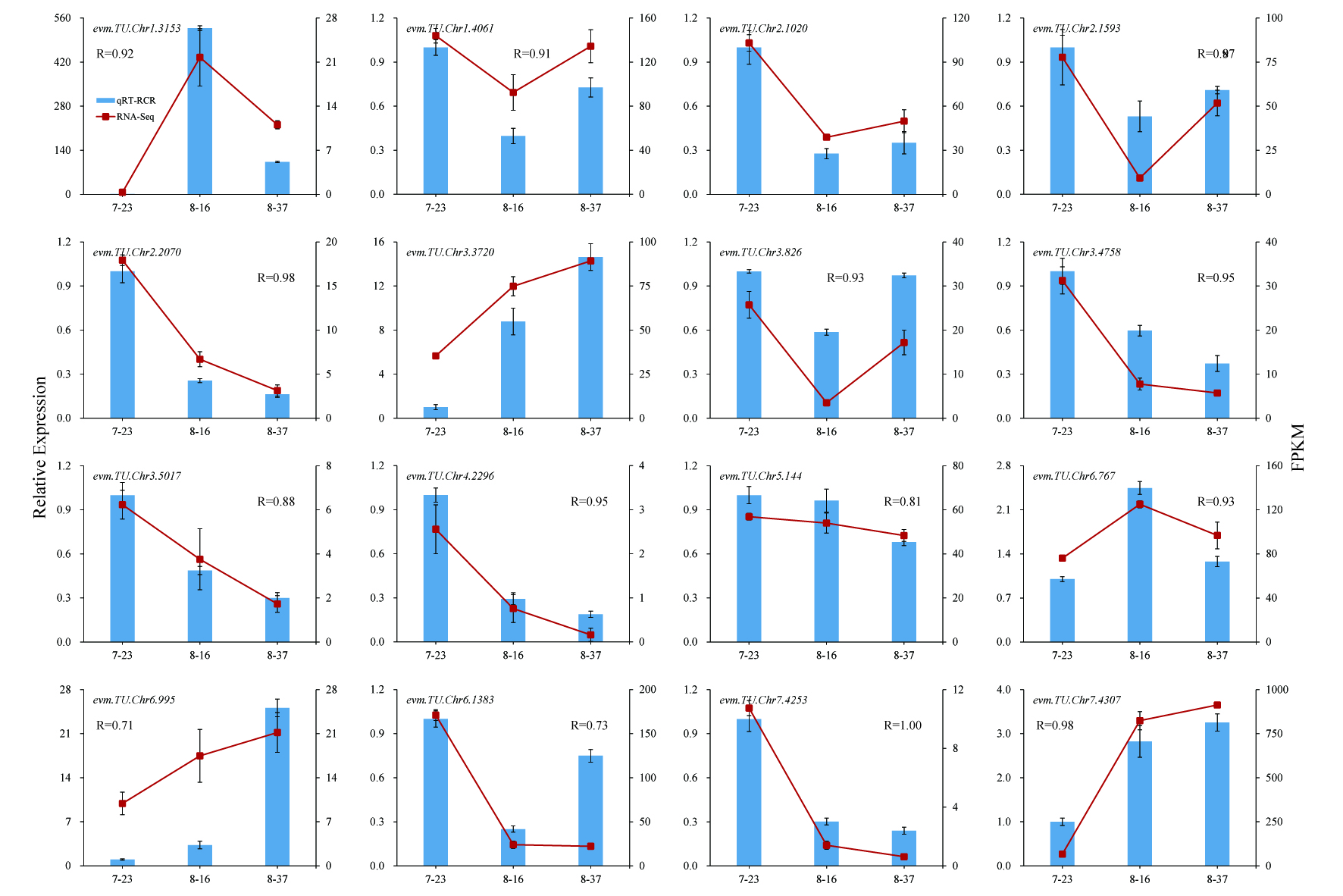

Supplement: Supplementary file 1 [file Image_1.jpeg]

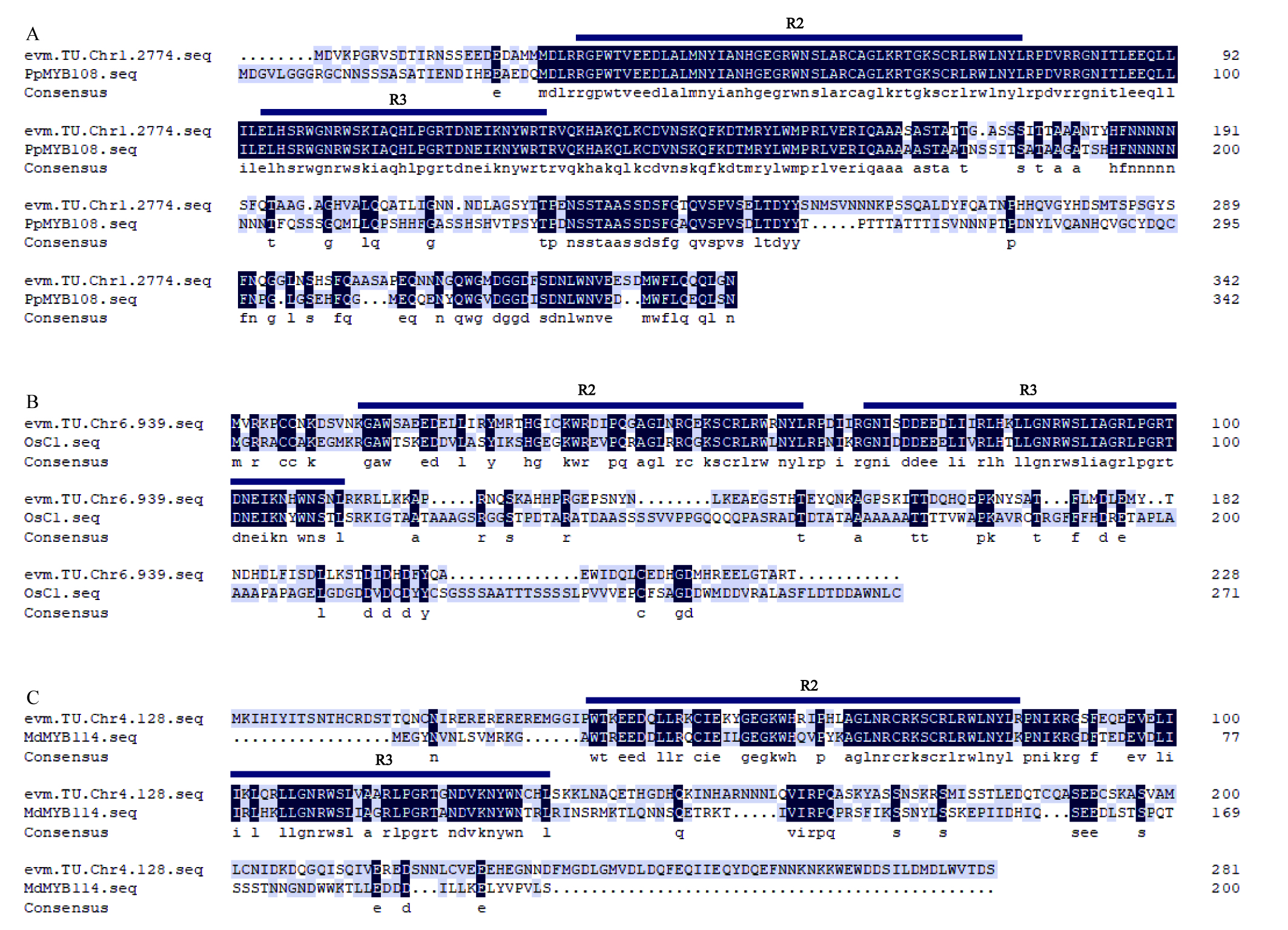

Supplement: Supplementary file 2 [file Image_2.jpeg]
